# A promising method for fast identification of microplastic particles in environmental samples: A pilot study using fluorescence lifetime imaging microscopy

**DOI:** 10.1016/j.heliyon.2024.e25133

**Published:** 2024-01-25

**Authors:** Maximilian Wohlschläger, Martin Versen, Martin G.J. Löder, Christian Laforsch

**Affiliations:** a Faculty of Engineering, Technical University of Applied Sciences Rosenheim, Hochschulstraße 1, 83024 Rosenheim, Germany; b Animal Ecology I and BayCEER, University Bayreuth, Universitätsstraße 30, 95440 Bayreuth, Germany

**Keywords:** Microplastics, Material identification, Environmental science, Fluorescence lifetime, FD-FLIM, Fluorescence microscopy

## Abstract

Microplastic pollution of the environment has been extensively studied, with recent studies focusing on the prevalence of microplastics in the environment and their effects on various organisms. Identification methods that simplify the extraction and analysis process to the point where the extraction can be omitted are being investigated, thus enabling the direct identification of microplastic particles. Currently, microplastic samples from environmental matrices can only be identified using time-consuming extraction, sample processing, and analytical methods. Various spectroscopic methods are currently employed, such as micro Fourier-transform infrared, attenuated total reflectance, and micro Raman spectroscopy. However, microplastics in environmental matrices cannot be directly identified using these spectroscopic methods. Investigations using frequency-domain fluorescence lifetime imaging microscopy (FD-FLIM) to identify and differentiate plastics from environmental materials have yielded promising results for directly identifying microplastics in an environmental matrix. Herein, two artificially prepared environmental matrices that included natural soil, grass, wood, and high-density polyethylene were investigated using FD-FLIM. Our first results showed that we successfully identified one plastic type in the two artificially prepared matrices using FD-FLIM. However, further research must be conducted to improve the FD-FLIM method and explore its limitations for directly identifying microplastics in environmental samples.

## Highlights

• Proof-of-principle for FD-FLIM measurement of microplastic particles in a soil matrix.
• Characterization of the spectral fluorescence behavior of HDPE, spruce and grass.
• Determination of the fluorescence lifetimes of HDPE, spruce and grass using FD-FLIM.
• Automated classification of different materials in FD-FLIM images.
• Possible detection of microplastic particles at least down to a size of 70 μm using FD-FLIM.

## Graphical abstract

**Figure:**
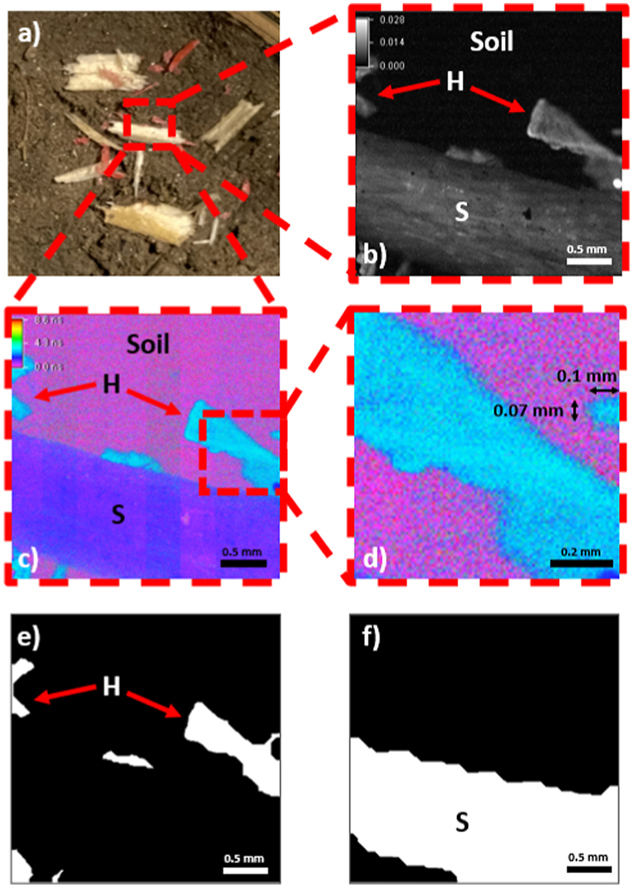

## 1 Introduction

Environmental pollution caused by plastic waste is a well-known global issue that increases the burden on society [1]. Several million tons of plastic enter the world's oceans annually [2,3], which poses a problem for the environment and ecosystems [[4], [5], [6], [7]]. Especially microplastics (MPs), small plastic particles <5 mm, have gained worldwide attention during the last two decades because freshwater and terrestrial environments are ubiquitously contaminated with them [[8], [9], [10]].

Although MPs are abundant in nature, their impact on the environment and human health remains unclear. Because the impact of MPs on ecosystem functions and organisms depends on the level of exposure and the material properties of the particles, accurately assessing MP contamination in terms of the number, polymer type, shape, and size is essential. However, the reliable analysis of MPs is challenging because it requires extensive sample preparation.

Currently, thermal extraction and desorption-gas chromatography/mass spectroscopy [11] or pyrolysis-gas chromatography/mass spectroscopy [12] are used to determine the mass-related proportion of MPs in an environmental sample. However, these methods do not provide data on particle size, which is important for an ecological risk assessment. Appropriate analytical tools and methods need to be applied to ensure a good ecological risk assessment because visual identification is extremely prone to bias, with error rates of up to 70 % [13,14].

The current state-of-the-art method for analyzing MPs in environmental samples comprises three major steps: 1) Sampling; 2) sample processing and extraction; and 3) identification and quantification [8]. Because environmental samples are typically characterized by an unfavorable target-to-non-target ratio (i.e., the amount of environmental matrix vastly exceeds the number of MPs), sample processing and the extraction of MP particles are essential prior to using analytical methods. Currently, the most commonly used analytical methods for precisely identifying MP particles extracted from environmental samples are Raman spectroscopy and Fourier-transform infrared (FTIR) spectroscopy [13]. MP particles that are ≤1 μm can be analyzed using Raman micro-spectroscopy with a non-negligible error [15]. By combining micro-FTIR spectroscopy with a focal plane array detector (FPA), particles in the range from 10 to 500 μm can be identified quickly [16]. Additionally, based on the spatial resolution of the FPA detector, analyzing MPs in sediments is possible using micro-FTIR spectroscopy if the samples are dry [17,18]. Attenuated total reflectance (ATR)-FTIR spectroscopy can be employed to analyze MP particles > 500 μm [19]. However, spectroscopic techniques have disadvantages in directly identifying MPs in original and undried environmental samples. FTIR spectroscopy is sensitive to water owing to its high absorption band. Thus, the superimposition of the IR spectrum of MPs renders their identification difficult. Identification using Raman micro-spectroscopy is difficult if organic impurities are present on the plastic surface, which can occur even after purification or if fluorescent pigments are added to the plastic. These contaminants can result in high fluorescence signals, thus superimposing the desired Raman spectra [20]. All currently available and reliable analytical techniques require efficient extraction and sample preparation to achieve a high purification rate and to identify the shape, size, and plastic type of the MP sample [20]. The preparation and purification of extracted samples of undesired secondary environmental particles are time-consuming and can be omitted if the MP can be identified directly on-site in the accompanying matrix [8]. Accordingly, more time-efficient measurement methods for the large-scale analysis of environmental samples without complex extraction steps are highly desirable, particularly concerning MP monitoring programs, as stipulated in the European Marine Strategy Framework Directive. Two imaging methods that potentially enable the rapid identification of MP directly in environmental matrices are time-domain and frequency-domain fluorescence lifetime imaging microscopy (TD-FLIM and FD-FLIM, respectively).

Langhals et al. [21] and Gies et al. [22] measured the fluorescence lifetime in TD and demonstrated that fluorescence lifetime can be used to identify plastics. Langhals et al. [21] investigated polystyrene, high-density polyethylene (HDPE), low-density polyethylene, polymethylmethacrylate (PMMA), polycarbonate (PC), ultra HDPE, polyethylene terephthalate (PET), and the technical polymers Luran (styrene, polyacrylonitrile copolymer), Delrin (polyoxymethylene), and Ultramid (polyamide with glass fiber) in terms of their punctual fluorescence decay time in the TD using a PicoQuant FluoTime 300 at an excitation wavelength of 405 nm. Gies et al. [22] investigated the polymers polypropylene, polytetrafluoroethylene, polyamide 6 (PA6), PA6.6, PC, PMMA, polyvinylidene fluoride, polyvinyl chloride (PVC), four natural materials (sea snail shell, piece of Mediterranean mussel, piece of cuttlebone of a cuttlefish, piece of sea grass), and quartz sand bought from a store. A neodymium-doped yttrium aluminum garnet laser was used as the excitation source, and a spectrometer and charge-coupled device (CCD) were used as the detection units. The results of both contributions showed that the identification and differentiation of plastics are possible, and plastics can be differentiated from non-plastic materials. Monteleone et al. [23] successfully demonstrated the high potential for identifying and characterizing six plastics, which included PA6, PET, acrylonitrile butadiene styrene, polylactide, polyphenyl ether (PPE), and polyurethane, using a TD-FLIM system. The plastics were shredded in a cryogenic swinging mill to generate the MPs required for the investigations. Fluorescence lifetimes of the generated MPs were measured using a Leica TGS SP8 FALCON system. The MP particles of PA6 and PPE could be differentiated within a single TD fluorescence lifetime image. However, the low fluorescence intensity of plastics resulted in a high integration time for determining the fluorescence lifetime, thus the measurement required a relatively long time.

Analogous to the fluorescence lifetime measurements in the TD, determining the decay time in the FD is possible [24]. Our previous research showed that the differentiation of PA, polyethylene, PET, and PVC plastics was possible using an FD-FLIM system [25]. A pco.flim FD-FLIM camera from Excelitas PCO GmbH was used in combination with a 488 nm excitation laser diode from Omicron-laserage GmbH. Using this experimental setup, fluorescence lifetime images were obtained and evaluated using Gaussian analysis. The results of Gaussian analysis showed that the differentiation and identification of the four polymers was possible by measuring the fluorescence lifetimes of the individual materials. Similarly, the differentiation of PA, PET, and PVC plastics from oak and spruce wood samples was possible by utilizing the measured fluorescence lifetime of the individual materials using the pco.flim system and Gaussian evaluation algorithm [26]. The investigations demonstrated the potential of the FD-FLIM measurement method to identify plastics and differentiate plastics from non-plastic materials, where the materials were always individually measured. However, FD-FLIM has yet to be evaluated for the direct identification of MP particles in environmental matrices.

As outlined above, the current sampling, extraction, and purification techniques necessary for identifying MPs in an environmental matrix using spectroscopic techniques are time-consuming. This study aimed to demonstrate for the first time whether MP particles could be directly detected in an artificially prepared environmental matrix and identified using FD-FLIM. We provide an overview of the standard measurement procedure for FD-FLIM, description of the four conducted experiments, and obtained initial results, which indicate that in principle, FD-FLIM has high potential for the detection of MPs in the environment. Furthermore, we discuss the necessary future investigations based on these preliminary results.

## 2 Theory

**Fig. 1:**
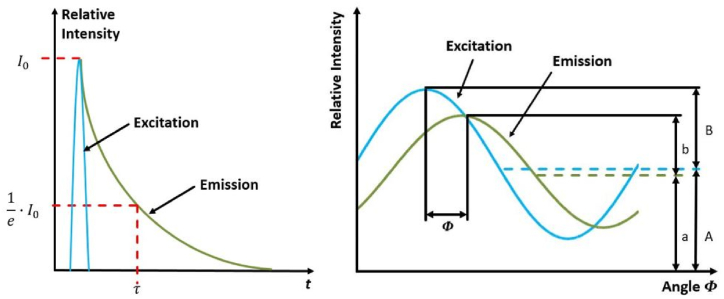
Left: Schematic representation of the fluorescence lifetime measurement in TD, with excitation (blue) and exponentially decreasing emission (green). Right: Schematic representation of the fluorescence lifetime measurement in FD, with excitation oscillation (blue) and harmonic phase-shifted, amplitude damped, and equivalent shifted fluorescence signal (green). (For interpretation of the references to color in this figure legend, the reader is referred to the Web version of this article.)

In principle, fluorescence lifetime can be determined using TD or FD fluorimetry [24]. In TD fluorimetry, the examined material is excited using a laser pulse of a defined wavelength that lasts a few nanoseconds (Fig. 1, left, blue curve). Excitation produces a wavelength-dependent Stokes shifted fluorescence signal. Once the excitation peaks, the fluorescence intensity attains its maximum *I*_*0*_, and the fluorescence intensity of the material exponentially decays (Fig. 1, left, green line). The fluorescence lifetime *τ* is the time after which the initial fluorescence intensity *I*_*0*_ has decayed to *I*_*0*_*/e* (Fig. 1, left). The fluorescence lifetime, which is in the nanosecond range, is material-specific and is commonly measured using a photoelectron amplifier in combination with a sensitive detector.

In contrast, fluorescence lifetime can be determined using FD fluorimetry, where the material is excited with a sinusoidally or rectangular modulated light oscillation with a defined modulation frequency *ω* (Fig. 1, right, blue oscillating curve). The modulated light oscillation in the FD represents the Fourier-transform of pulsed laser excitation. The oscillating excitation causes a harmonic fluorescence signal, phase-shifted by the angle *φ*. In addition, the fluorescence signal is amplitude-damped (*b*) and equivalent-shifted (*a*) to the excitation signal (*B*, *A*) (Fig. 1, right, green oscillation). By measuring the phase shift *φ*, the phase-dependent fluorescence lifetime *τ*_*PH*_ can be calculated using Equation (1).

(1) $$\tau_{PH}=\frac{\tan\left(\varphi \right)}{\omega }$$

Furthermore, calculation of the modulation index *M* is possible with the measured amplitudes (*A*, *a*) and equivalent shifts (*B*, *b*) using Equation (2).

(2) $$M=\frac{\left(b/a\right)}{\left(B/A\right)}$$

Using the calculated modulation index *M* and defined modulation frequency *ω*, the modulation-dependent fluorescence lifetime *τ*_*M*_ can be derived using Equation (3).

(3) $$\tau_{M}=\frac{\sqrt{\frac{1}{M^{2}}-1}}{\omega }$$

Based on the theory of FD fluorimetry, the areal measurement of fluorescence lifetime was possible using FD-FLIM. The fluorescence oscillation was measured using an FD-FLIM camera system that comprised a photodiode (pixel) matrix, where every pixel could measure fluorescence intensity *I*, phase shift *φ*, and modulation index *M* (using *a*, *A* and *b*, *B*). Using these three measured parameters, calculations of pixelwise phase- and modulation-dependent fluorescence lifetimes (*τ*_*PH*_ and *τ*_*M*_, respectively) were possible. Thus, the measurements resulted in a picture stack that contained images of fluorescence intensity, phase shift, modulation index, and phase- and modulation-dependent fluorescence lifetimes. The measured image stack could be used to identify different materials in a single image.

## 3 Materials and methods

Four experiments were conducted to demonstrate the potential of FD-FLIM for directly identifying MPs in an environmental matrix.

(I) Spectral fluorescence analysis of spruce, grass, and red HDPE was performed to determine the spectral fluorescence behavior of the materials.
(II) FD-FLIM measurements of the same homogeneous samples were performed to build a database to classify the three materials in a single fluorescence lifetime image.
(III) FD-FLIM measurements of an artificial inhomogeneous matrix containing high ratios of spruce, grass, and HDPE were performed, and the different materials were classified based on previously measured data.
(IV) FD-FLIM measurements of an artificial inhomogeneous matrix containing low ratios of spruce, grass, and HDPE were performed in a natural soil background, which focused on the direct identification and classification of red HDPE particles in an environmental matrix using the FD-FLIM method

## 4 Experimental details for the spectral analysis and measurement of the fluorescence lifetime of plastics and MPs

**Fig. 2:**
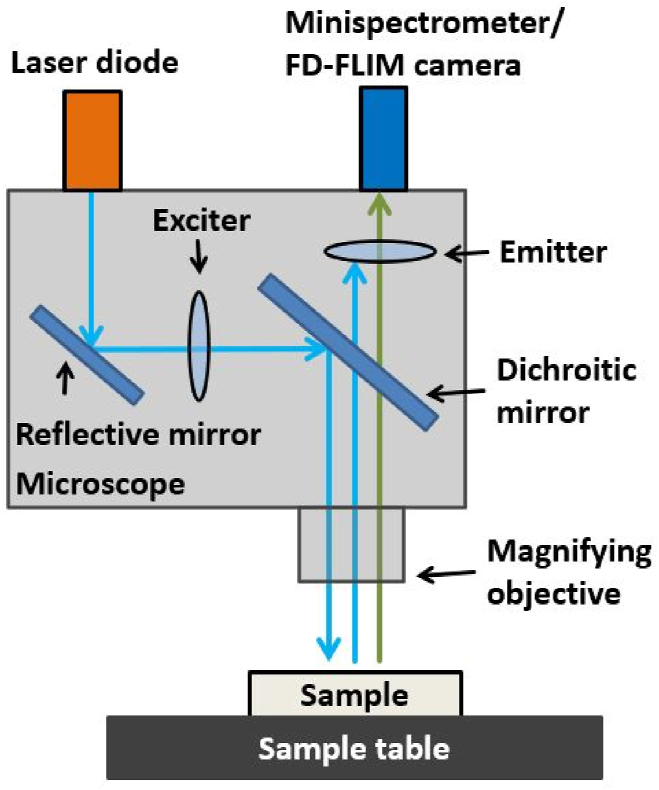
Experimental setup for the spectral fluorescence analysis/fluorescence lifetime measurements containing a laser diode, Mini-spectrometer/FD-FLIM camera system, and optical filters (exciter, emitter) all assembled on the microscope.

Schematically displayed in Fig. 2, the experimental setup was assembled using a laser diode PhoxX 488 from Omicron, a Mini-spectrometer from Hamamatsu, pco.flim camera from PCO AG, probe station EPS150FA from Cascade Microtech, and optical filters from AHF Analysentechnik. The laser diode provided an excitation wavelength of 488 nm and power of 200 mW, and the Mini-spectrometer was used to measure the spectral fluorescence properties. The CCD image sensor in the Mini-spectrometer had 2048 pixels, where every pixel detected a different wavelength in the range of 200–1000 nm at a spectral resolution of 1 nm. To measure the fluorescence lifetime, the Mini-spectrometer was disassembled from the microscope, and an FD-FLIM camera was connected. The pco.flim camera measured fluorescence decay times in the range of 100 ps to 100 μs with a dynamic range of 10 bits [27]. The complementary metal oxide semiconductor image sensor in the pco.flim had a size of 5.6 mm × 5.6 mm and a resolution of 1004 × 1008 pixels. Accordingly, a pixel had a height of 5.6 μm and width of 5.6 μm. Every pixel represented an independent location-dependent measurement of fluorescence intensity, phase shift, and modulation index. The probe station EPS150FA from Cascade Microtech included a microscope (PSM1000 from Motic) with magnifications at 20 × , 10 × , or 2 × . If the magnification of the microscope was set to 20 × , images with a size of 0.28 mm × 0.28 mm (pixel size: 0.28 μm × 0.28 μm) could be taken. Similarly, images of 0.56 mm × 0.56 mm (pixel size: 0.56 μm × 0.56 μm) using 10 × magnification or 2.8 mm × 2.8 mm (pixel size: 2.8 μm × 2.8 μm) using 2 × magnification could be captured. A laser diode, Mini-spectrometer, and pco.flim camera were assembled to the microscope. Three optical filters from AHF Analysentechnik that could be inserted into the microscope were selected for the experiments. The exciter was a laser cleanup filter that narrowed the bandwidth of the laser diode along the excitation light path. Two optical filters were used as emitters for the experiments. A long-pass (LP) filter with a cut-on wavelength of 500 nm was used to detect the entire fluorescence spectrum. A band-pass (BP) filter with cut-on and cut-off wavelengths of 495 and 550 nm, respectively, was used to detect only the fluorescence signal in the wavelength range of 495–550 nm.

## 5 Sample preparation

Grass, spruce, natural soil, and red HDPE were investigated to demonstrate the potential of the FD-FLIM method in directly identifying MPs in environmental samples. Grass was collected from the lawn at Technical University Rosenheim and cut into pieces that were approximately 2 cm long and 3 mm wide. Soil was collected from the grassland at Technical University of Rosenheim at a depth of 5 cm and represented standard grassland soil. However, it was not further characterized in terms of clay, humus, organic compound, or water contents. Monitoring the effects of these parameters and the different soil types should be investigated in the future. A sliver of spruce with a length of 2 cm and width of 5 mm was obtained from a block of dry spruce wood. HDPE was cut from a Euronorm E2 box and its triangular shape was 1.5 cm in height and 1.5 cm in width. Experiments I and II (i.e., single-material measurements) were performed using individual samples (Fig. 5a). Two sample arrangements were prepared to simulate real-world circumstances (Fig. 5, Fig. 6a). As shown in Fig. 5a, three large samples of spruce, grass, and HDPE were positioned next to each other to determine the impact of different fluorescence lifetimes on each other using the FD-FLIM method (Experiment III.) The actual area of the image is marked in red. Sample preparation for Experiment IV represented the arbitrary placement of small slivers of red HDPE plastic next to the spruce splinters on the soil (Fig. 6a). The spruce and HDPE were cut into small pieces using a scalpel. The HDPE slivers were 1–2 mm in size and the spruce splinters were approximately 5 mm long and 1 mm thick. The FD-FLIM measurements of the small samples in Fig. 6a demonstrated the size limitations of the FD-FLIM method for directly identifying MPs in an environmental sample.

## 6 Experimental procedures and data evaluation

In this section, the experimental procedures and data evaluation algorithm for the spectral fluorescence (I), single-material FD-FLIM (II), and multi-material FD-FLIM measurements (III and IV) are explained.

### 6.1 Experimental procedure to measure the fluorescence spectra (I)

A Mini-spectrometer was connected to a microscope for the spectral fluorescence analysis of each material. The laser power was adjusted to a maximum of 200 mW, the microscope magnification of 10 × was selected, and the Mini-spectrometer exposure time was set to 2 s. Hamamatsu evaluation software was used to measure the fluorescence spectra of each material. Two different measurements were performed on five different sites of HDPE, spruce, and grass: one with an optical LP filter and the other with an optical BP filter as the emitter. Limiting the fluorescence spectrum affected the fluorescence lifetime that was measured using the FD-FLIM system. Therefore, determining the parts of the fluorescence spectrum that are blocked by an optical BP filter is essential. A background measurement against air was performed to obtain the noise in the measurement setup. Using this setup, the fluorescence spectra (I) of the spruce, grass, and HDPE were measured. Spectral data were stored in a comma-separated file for further processing and analysis.

### 6.2 Data analysis of spectral fluorescence measurements (I)

The fluorescence spectra were imported into MATLAB for evaluation. After subtracting the measured background spectrum from the fluorescence spectra of each material to eliminate noise, five measured spectra for each material were averaged and interpolated using an interpolation step size of 0.01 nm. The interpolated spectra were plotted and the intensity was displayed as a function of wavelength. In addition, the maxima and corresponding wavelengths were obtained from the interpolated spectra. Finally, the Stokes shift was calculated by subtracting the excitation wavelength 488 nm from the obtained wavelengths of the corresponding maxima.

### 6.3 Experimental procedure of single-material FD-FLIM measurements (II)

The FD-FLIM camera system was connected to a microscope to measure fluorescence lifetime. The camera was referenced to measure the fluorescence lifetime, and NIS elements (Nikon) were used for the reference procedure and fluorescence lifetime measurements. The optical output power was set to its maximum. A reference slide from Starna Scientific with a standardized fluorescence lifetime of 3.75 ns was placed on the stage under the microscope, and the magnification was adjusted to 10 × . The modulation frequency was set to 30 MHz, and the appropriate exposure time was adjusted in the range of 68 %–72 % of the maximum exposure time of the camera. The camera was referenced using these configurations.

After referencing, grass, spruce, and HDPE were placed on the sample stage and FD-FLIM measurements of each material were conducted. The images of the individual materials contained five layers of 1004 × 1008 fluorescence intensity, phase shift, modulation index, and phase- and modulation-dependent fluorescence lifetimes. Only the fluorescence intensity and phase-dependent fluorescence lifetime images were analyzed. Accordingly, the measurement of the phase-dependent fluorescence lifetime image resulted in 1004 × 1008 data points, that is, approximately 1 million fluorescence lifetimes were measured per material, which was sufficient for classifying multiple materials in a single fluorescence lifetime image. The resulting TIF (tagged image file) stack containing five TIF images of the layers was saved and evaluated using the algorithm described in section 6.4.

### 6.4 Data analysis of single material FD-FLIM measurements (II)

Evaluation of a single-material fluorescence lifetime image was conducted as previously described [25]. First, TIF images were imported into the MATLAB workspace. Next, a histogram of the 1004 × 1008 measured fluorescence lifetimes was created, which displayed the absolute frequency of each fluorescence lifetime in the image. Using Gaussian analysis, the expectation value and standard deviation were calculated, enabling numerical identification. In addition, graphical identification was possible by plotting the resulting normalized Gaussian curves.

### 6.5 Experimental procedure of multi-material FD-FLIM measurements (III & IV)

Referencing and multi-material measurements were also performed using NIS Elements software. The experimental procedure for the multi-material FD-FLIM measurements was similar to that for the single-material FD-FLIM measurements. After the measurement setup was referenced using the calibration slide and modulation frequency of 30 MHz, the sample (Fig. 5a, right) was investigated at a magnification of 10 × . The exposure time was set to 400 ms and measurements were performed. The resulting five-image TIF stack was stored for further data processing.

The sample in Fig. 6a was examined using 2 × magnification and a modulation frequency of 30 MHz. As the magnification was changed, the experimental setup was recalibrated using the calibration procedure described above. After calibrating the measurement setup, the exposure time for fluorescence lifetime measurements was set to 1500 ms. For identifying MPs in the environment, a lower magnification of 2 × was chosen because larger areas could be analyzed in one measurement in the later intended application. Measurements were performed, resulting in a TIF stack that was stored for evaluation.

### 6.6 Data analysis of multi-material FD-FLIM measurements (III & IV)

**Fig. 3:**
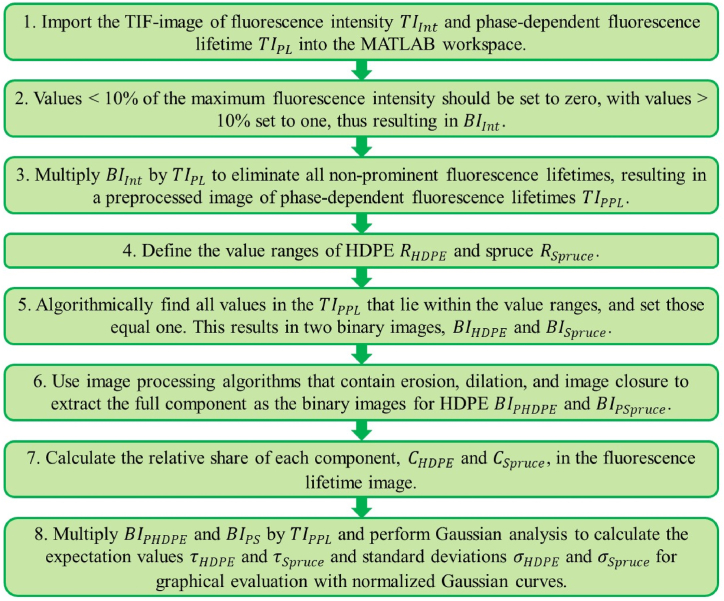
Flow chart for the data evaluation of multi-material FD-FLIM measurements.

The steps for data analysis are shown in the sequence diagram (Fig. 3). First, the TIF images of the fluorescence intensity $TI_{\mathrm{Int}}$ and phase-dependent fluorescence lifetime $TI_{PL}$ were imported into the MATLAB workspace as a matrix of 1004 × 1008 values.

The TIF-image of the fluorescence intensity $TI_{\mathrm{Int}}$ was rescaled by setting all intensities that had a value < 10 % of the maximum fluorescence intensity present in the image to zero. Additionally, all values > 10 % of the maximum fluorescence intensity were set to one. The resulting binary image $BI_{\mathrm{Int}}$ was multiplied by the TIF image of the phase-dependent fluorescence lifetime $TI_{PL}$ as a mask to eliminate all fluorescence decay times that had no or almost no influence on the image or that represented noise. This process resulted in a preprocessed phase-dependent fluorescence lifetime image $TI_{PPL}$ where only the prominent fluorescence lifetimes were displayed.

To extract the different components in the preprocessed phase-dependent fluorescence lifetime image $TI_{PPL}$, the two value ranges for HDPE ($R_{HDPE}=3.52\pm 3\times 0.21\;ns$) and spruce ($R_{Spruce}=1.40\pm 3\times 0.12\;ns$) were defined as upper and lower boundaries. These values were derived from the previous single-material FD-FLIM measurements from II. A search algorithm found all the values in the preprocessed phase-dependent fluorescence decay time image $TI_{PPL}$ within the defined ranges of HDPE and spruce. The corresponding pixel values were set to one and stored in two new binary images, where in a binary image one was represented as white and zero as black for HDPE $BI_{HDPE}$ and spruce $BI_{Spruce}$.

Four image processing operations were performed based on these binary images. The first operation was digital erosion with a rectangular mask to eliminate all the incorrectly counted noise pixels. In the second and third steps, digital dilation and digital image closure were executed with a disk mask to set all pixels to one that were incorrectly counted as zero because they were outside the value range but within the material boundaries of the image. In the fourth and final steps of image processing, digital erosion was performed again for edge smoothing purposes. Two processed binary images contained the extracted components for HDPE $BI_{PHDPE}$ and spruce $BI_{PSpruce}$.

Using these images, the relative share of the two components, $C_{HDPE}$ and $C_{Spruce}$, presented in the image was calculated by dividing the number of pixels equal to one by the total number of pixels, that is, 1004 × 1008. Furthermore, the two binary images, $BI_{PHDPE}$ and $BI_{PSpruce}$, were multiplied by the preprocessed phase-dependent fluorescence decay time image $TI_{PPL}$, resulting in two phase-dependent fluorescence lifetime images: $TI_{PLHDPE}$ for HDPE and $TI_{PLSpruce}$ for spruce. Gaussian analysis was performed with these two images to calculate the corresponding expectation values $\tau_{HDPE}$ and $\tau_{Spruce}$ and standard deviations $\sigma_{HDPE}$ and $\sigma_{Spruce}$. Normalized Gaussian curves were created to graphically evaluate the results.

## 7 Results

### 7.1 Results of the spectral fluorescence measurements (I.)

**Fig. 4:**
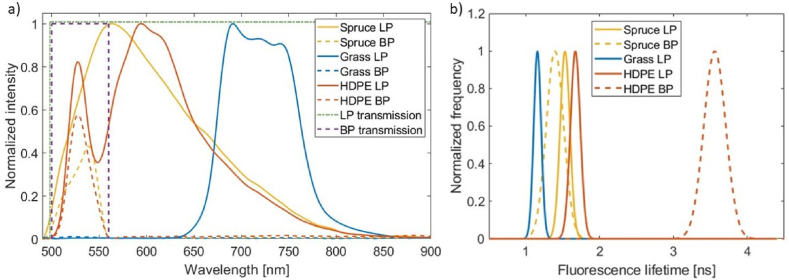
a) Normalized fluorescence intensities as a function of the wavelength of grass, spruce, and HDPE with ideal transmission functions of the BP (purple dashed line) and LP (green stroke-dotted line) filters. Measurements of the materials using the LP filter are plotted with straight lines and the fluorescence spectra measured using the BP filter are plotted as dashed lines. b) Normalized histograms of the Gaussian analysis for the FD-FLIM measurements using the optical BP (dashed lines) and LP filters (straight lines) of spruce, grass, and HDPE. (For interpretation of the references to color in this figure legend, the reader is referred to the Web version of this article.)

Results of the normalized spectral fluorescence measurements are shown in Fig. 4a. By measuring the fluorescence spectra with an LP filter, the red HDPE plastic showed two fluorescence maxima: one at 528 nm and the other at 594 nm. The first maximum at 528 nm corresponds to the polymer because plastics generally exhibit only a small Stokes shift in the range of 20–30 nm [21]. The filler, additive, or dye added to the polymer caused a more prominent fluorescence maximum at 594 nm. The measured fluorescence spectra of grass using the optical LP filter exhibited fluorescence maxima of grass from 680 to 770 nm, which were caused by the excitation of chlorophyll *a* present in leaves [28]. Spruce has a fluorescence maximum at 564 nm, which is primarily caused by lignin, the main component of wood cell walls [29].

**Fig. 5:**
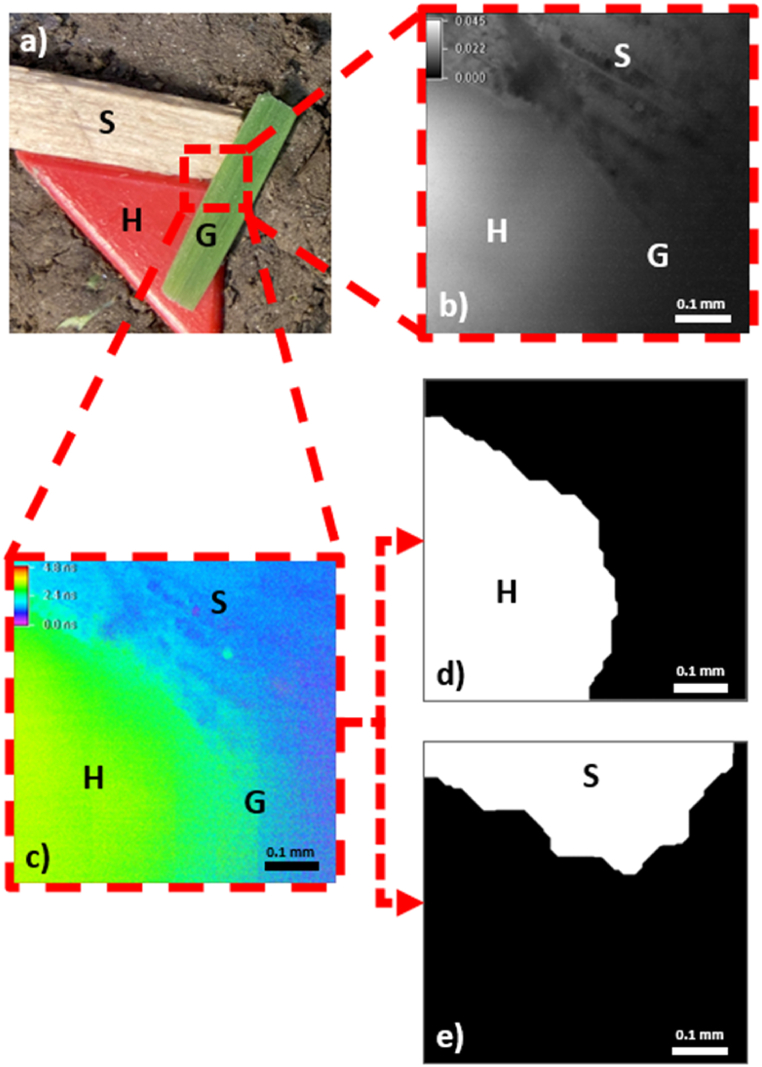
a) Prepared pieces of HDPE plastic (H), spruce (S), and grass (G) on soil. b) Fluorescence intensity images of S, H, and G taken with a magnification of 10 × . c) Phase-dependent fluorescence lifetime images of S, H, and G taken with a magnification of 10 × . d) and e) Binary images $BI_{PSpruce}$ of S and $BI_{PHDPE}$ of H taken and processed at a magnification of 10 × .

**Fig. 6:**
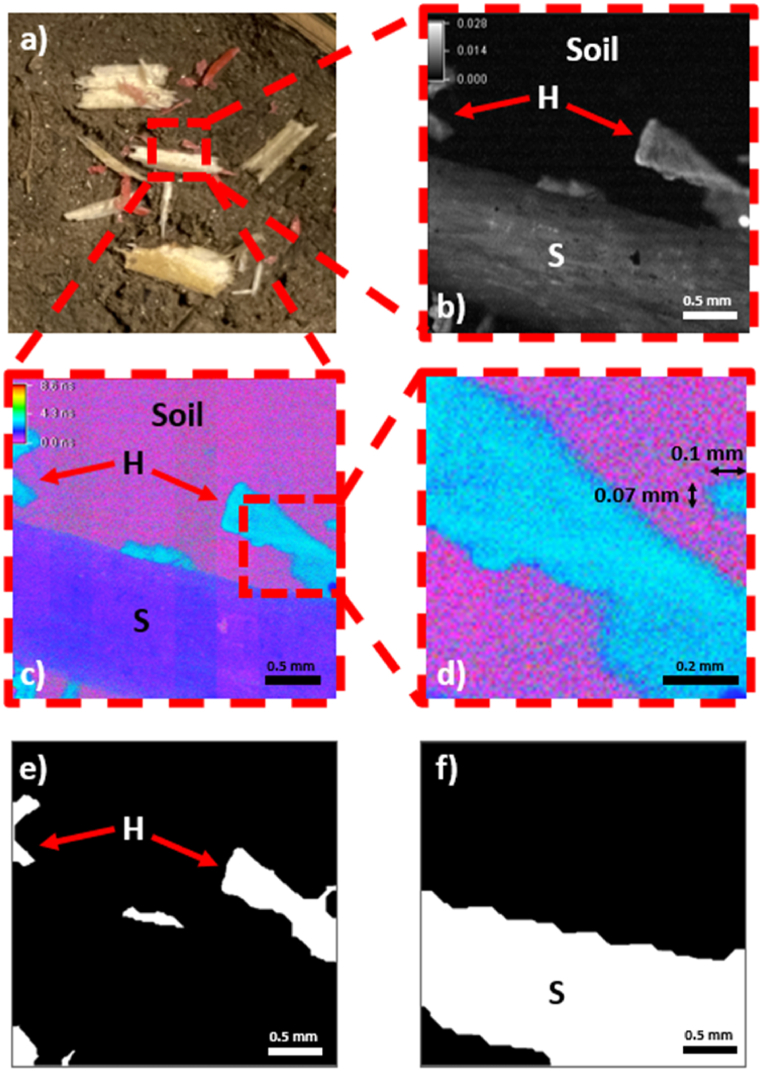
a) Prepared chips of HDPE plastic (H) and spruce (S) on soil. b) Fluorescence intensity images of S and H on soil taken with a magnification of 2 × . c) Fluorescence lifetime images of S and H on soil taken with a magnification of 2 × , where the pixel size is 2.8 × 2.8 μm. (d) Detailed view of the smallest H particle in the fluorescence lifetime image (c) including the measured size. Binary images $BI_{PHDPE}$ of the extracted H data (e) and $BI_{PS}$ of extracted S data (f) on soil taken and processed at a magnification of 2 × .

When the optical BP filter was used instead of the LP filter for measuring the fluorescence spectra, the fluorescence intensities of HDPE and spruce decreased. In addition, the second maximum of the fluorescence from HDPE, which originated from the red dye of HDPE, was blocked such that only the polymer fluorescence maximum at 528 nm was detected. Furthermore, the fluorescence signal of grass was entirely blocked by the BP filter. Blocking by the BP filter is advantageous for investigations with the FD-FLIM camera system because the grass signal is blocked and spruce and HDPE are distinguished.

The obtained values of the fluorescence maxima and calculated values of the Stokes shift are listed in Table 1, which summarizes the results from Fig. 4a.

**Table 1:** Results of the spectral fluorescence measurements of grass, HDPE, and spruce.

Material	Optical filter	Wavelength of the maximum [nm]	Stokes shift [nm]
Grass	LP	691	203
		719	231
		741	253
Grass	BP	–	–
HDPE	LP	528	40
		594	106
HDPE	BP	528	40
Spruce	LP	563	75
Spruce	BP	539	51

### 7.2 Results of the single material FD-FLIM measurements (II.)

The calculated mean values (expectation values) and standard deviations of the fluorescence lifetime images are presented in Table 2. As shown, identifying plastics is more difficult with an optical LP filter than with a BP filter. Distinguishing between the spruce and HDPE was impossible within one standard deviation using the optical LP filter. Moreover, grass has a low fluorescence lifetime, similar to that of spruce and HDPE. The measurements with the optical BP filter indicated easier differentiation than those with the optical LP filter. However, because the fluorescence lifetime of grass was avoided, HDPE and spruce could be clearly distinguished. The different lifetime values of HDPE can be explained by the dominance of the 594 nm maximum in the LP measurement, which was filtered through the BP measurement. The 3.52 ns fluorescence lifetime was maximum at 528 nm. Therefore, an optical BP filter was chosen to differentiate multi-material identification in a single fluorescence lifetime image. Furthermore, two ranges for fluorescence decay times were defined using single-material FD-FLIM measurements. The upper boundary was set as the expectation value plus three times the standard deviation, and the lower boundary was set as the expectation value minus three times the standard deviation. For the materials, the ranges of $R_{HDPE}=3.52\pm 3\times 0.21\;ns$ and $R_{\text{Spruce}}=1.40\pm 3\times 0.12\;\text{ns}$ are defined.

**Table 2:** Calculated expectation value and standard deviation assuming a Gaussian normal distribution from the fluorescence lifetimes of grass, HDPE, and spruce, measured with the optical LP and BP filters.

Material	Fluorescence lifetime measured with the optical LP filter (in ns)	Fluorescence lifetime measured with the optical BP filter (in ns)
Grass	1.15 ± 0.08	–
HDPE	1.68 ± 0.07	3.52 ± 0.21
Spruce	1.54 ± 0.07	1.40 ± 0.12

The same result was obtained using graphical evaluation of the single-material measurements (Fig. 4b). Identification and differentiation were not possible using an optical LP filter (straight lines). In contrast, the measurements with the BP filter (dashed lines) clearly differentiated spruce and HDPE.

### 7.3 Result of the multi-material FD-FLIM measurement (III.)

Distinguishing the materials from each other (Fig. 5b) was difficult in the image of the fluorescence intensity captured by the pco.flim camera. A shadow of grass was observed (right) where the optical BP filter blocked the fluorescence signal. The spruce structure was visible on the upper side, and HDPE was visible on the lower left side.

If the fluorescence decay time image was plotted as a false color image, the different materials were distinguished (Fig. 5c). Grass on the right side appeared as noise, while spruce (shown in blue) had a faster fluorescence decay time than HDPE (shown in green).

Using the proposed image-processing algorithm, spruce and HDPE could be clearly distinguished from each other based on their specific fluorescence lifetimes. Fig. 5d and e show the binary extracted pictures of HDPE and spruce, respectively.

The expectation values and standard deviations of the fluorescence lifetimes of spruce and HDPE were calculated based on the extracted pixels belonging to the respective materials (Table 3). The corresponding pixel count was normalized by the total number of pixels and is expressed as a relative share in Table 3. The calculated decay times for spruce and HDPE collected from one image were within the range of the fluorescence lifetimes measured in the single-material FD-FLIM measurements (Table 2). Because the image processing time was approximately 2 s and the exposure time was set to 400 ms, a total of 2.4 s was required to identify and differentiate HDPE, spruce, and grass. Thus, identifying the materials was possible within a standard deviation using multi-material measurements in a single image at a magnification of 10 × .

**Table 3:** Fluorescence lifetimes and relative shares from the extracted components of HDPE and spruce.

Material	Fluorescence lifetime [ns]	Relative share [%]
Spruce	1.56 ± 0.52	25
HDPE	3.24 ± 0.60	41

### 7.4 Result of the multi-material FD-FLIM measurement (IV.)

To mimic an arbitrary sample placement in nature, the arrangement of small HDPE and spruce chips on soil were examined using 2 × magnification, as shown in Fig. 6a the 10 × magnification measurements, the individual materials could not be distinguished by fluorescence intensity (Fig. 6b).

HDPE and spruce are clearly distinguishable in the plotted, false color fluorescence lifetime image shown in Fig. 6c. Here, the fluorescence lifetime of HDPE is light blue, while that of spruce is dark blue. The soil is displayed as violet noise.

Fig. 6d shows a detailed view of the smallest particles detected in the fluorescence lifetime image at high magnification. If the width and height of a particle are measured using the number of pixels with NIS Elements, then the size of the particle can be calculated. At 2 × magnification, the pixel size was 2.8× 2.8 μm, which indicates that the particle in the image has a height of 70 μm and width of 100 μm (25 pixels and 35 pixels, respectively). Thus, particles can be identified at a size ≤70 μm using a magnification of 2 × if the fluorescence intensity of the particle is high enough. Given a total number of 10^6^ pixels of the imaging sensor and minimum detectable particle size of 25 × 25 pixels, the minimum detectable size corresponds to a relative share of 0.06 %, where the relative share describes the areal proportion of the material seen in the image (Fig. 6d).

Using the evaluation algorithm, two binary images, $BI_{PHDPE}$ for HDPE and $BI_{PSpruce}$ for spruce, were calculated (Fig. 6e and f). The fluorescence lifetimes were obtained and the normalized pixel counts of the fluorescence lifetimes are presented as relative shares in Table 4.

**Table 4:** Fluorescence lifetimes and concentrations from the extracted components of HDPE and spruce.

Material	Fluorescence lifetime [ns]	Relative share [%]
Spruce	1.23 ± 0.47	38
HDPE	2.97 ± 0.50	7

The fluorescence lifetimes differed, with 38 % and 7 % of pixels in the images counted as spruce and HDPE, respectively. The two materials were identified within one standard deviation and could be identified within one standard deviation using the values of the single-material fluorescence lifetime measurements shown in Table 2. The processing time of the evaluation algorithm was approximately 2 s. By adding an exposure time of 1.5 s to the image processing time, the identification of MP HDPE particles required approximately 3.5 s.

## 8 Discussion and conclusion

Spectroscopic methods are unsuitable for directly identifying MPs in environmental samples due to matrix interference. To eliminate these disadvantages and achieve a high purification rate, precise extraction and sample preparation must be performed. Otherwise, identifying the shape, size, and plastic type of the MP is hindered [20]. Because the extraction and preparation of the samples is time-consuming, appropriate and rapid imaging methods must be investigated to simplify the purification process and enable the direct identification of MP particles.

Fluorescence lifetime measurements in the TD show significant potential for identifying plastic types and distinguishing plastics from organic materials [21,22]. In addition, Monteleone et al. [23] demonstrated that identification utilizing the fluorescence lifetime is possible with a significance of 94.55 %. Moreover, they reported that differentiation and identification of the MP types PA6 and PPE are possible in the range of 100 μm by TD-FLIM using a laser scanning system. These studies concluded that the fluorescence decay time with TD-FLIM could provide a sensitive and straightforward identification system for MPs in the environment. However, TD fluorescence lifetime measurements involve photon-integration processes that may require a long time if the fluorescence intensity emitted by the examined material is low. In addition, the fluorescence lifetimes of the plastic types measured in the TD are not comparable to our results. In the TD, only the fluorescence lifetime of a single fluorescence wavelength is measured, while in our FD-FLIM measurements, the fluorescence lifetime of the complete fluorescence spectrum (optical LP filter) or the fluorescence lifetime of a part of the fluorescence spectrum (optical BP filter) was measured. Because part of the fluorescence spectrum in which the fluorescence lifetime is measured influences the fluorescence lifetime, comparing the fluorescence lifetimes was not reasonable [24].

In previous investigations using FD-FLIM, we demonstrated that the method is rapid and has a reasonable integration time. Our research demonstrated that different plastic types could be identified and differentiated [25]. We also demonstrated that distinguishing wooden oak and spruce types from plastic types was possible [26]. Thus, FD-FLIM was used for the current investigation because it is an areal measurement method that does not require sample scanning and has a faster integration time than TD-FLIM. Previous investigations using FD-FLIM have shown that the differentiation of plastics from wood is possible using an optical LP filter. Therefore, the next step was to investigate the direct and rapid identification of MPs in an artificially prepared environmental matrix comprising grass, soil, and spruce. In the first step, the spectral fluorescence behaviors of red HDPE, spruce, and grass were analyzed using different optical filters to determine the effect of blocked spectral regions on the fluorescence lifetime. Considering these spectral results, FD-FLIM measurements were performed.

The results of the spectral measurements using the laser and Mini-spectrometer showed that it was possible to identify all three materials. However, our results cannot be generalized because plastics often exhibit similar fluorescence spectra. For example, fluorescent markers are used by researchers to distinguish between plastic types using fluorescence spectroscopy [30]. In addition, it was determined that an optical BP filter could completely block the fluorescence emitted by grass in the range of 680–770 nm [28], which is advantageous for FD-FLIM measurements because only wood and plastic have to be differentiated from soil. Here, HDPE showed two fluorescence maxima, one at 528 nm and one at 594 nm, which were caused by the plastic and red dyes, respectively. The optical BP filter was advantageous because the fluorescence of the red dye was blocked and only the fluorescence of the plastic was detected by the FD-FLIM camera.

Differentiating the materials with an optical LP filter was difficult using the single-material FD-FLIM measurements. In our previous study [25,26], an optical LP filter was used to obtain the fluorescence lifetimes of wood and plastic. Consequently, the fluorescence lifetimes determined in the current study using an optical BP filter were not comparable. Using an optical BP filter, HDPE and spruce were identified and distinguished. The optical BP filter blocked the fluorescence response of grass and red dye in HDPE; therefore, no fluorescence decay time was measurable by the FD-FLIM camera system. By blocking the fluorescence response from the red dye of HDPE, the fluorescence lifetime became slower, which enabled the definite identification and differentiation of other materials. This blocking confirmed that suppressing the fluorescence signal from grass and the red dye of HDPE was advantageous for multi-material FD-FLIM measurements because only HDPE must be identified and differentiated from spruce.

Owing to the results obtained from spectral fluorescence (I) and single-material fluorescence lifetime measurements (II), an optical BP filter was used for multi-material measurements (III and IV). Using single-material measurement data, an algorithm was successfully applied to identify individual materials in fluorescence decay time images. Using this algorithm in combination with typical image processing methods, we extracted the individual components using 10 × and 2 × magnification. The shape and size of the MP particles were identified with binary processing using this algorithm. In addition, the relative shares were calculated for the individual components present in the fluorescence lifetime image.

Investigations using 10 × magnification showed that the differentiation and identification of HDPE and spruce were possible. The fluorescence signal of the grass was entirely blocked and appeared as noise in the fluorescence lifetime images. When the exposure time was set to 400 ms, the algorithm required approximately 2 s for the identification and calculation of the relative share. Therefore, the rapid differentiation and identification of large spruce, HDPE, and grass were possible in 2.4 s using FD-FLIM.

The investigation of MP HDPE particles and spruce splinters on soil using 2 × magnification demonstrated that MP particles could be identified in a larger measurement area. Furthermore, the pixel-relative share was calculated, providing information on the amount of identified material present in the image. The exposure time was set to 1.5 s, and the evaluation time of the algorithm was approximately 2 s, resulting in a total time of 3.5 s. Image processing and relative share calculations successfully demonstrated that the two materials were clearly distinguishable and identified within one standard deviation.

If the pixel size and magnification are known, the width and height of the particle as pixel values can be used to calculate the particle size. Thus, the most minor HDPE particle present in the image is measured, resulting in a height of 70 μm and width of 100 μm. Additionally, an HDPE particle with a pixel size of 25 × 25 can be clearly identified, and a concentration of 0.06 % is detected in the image. If the size of 25 × 25 pixels in which particles can be identified is transferred to the magnifications of 10 × and 20 × , it is possible to identify particles of 14 μm (10 × ) and 7 μm (20 × ). Further systematic research using different MP sizes (e.g. HDPE in the sizes of 100, 50, 25, and 10 μm) is required to determine the limitations of the FD-FLIM method.

In conclusion, this first study and its preliminary results demonstrated the identifiability of HDPE as an MP. Moreover, distinguishing it from spruce, grass, and soil demonstrated the potential of the FD-FLIM method to quickly identify MPs in the environment. However, given the vast variety of plastics in the environment, this method must be confirmed for different plastic types. Spruce and grass are also not the only environmental materials that must be characterized. These materials were chosen for the investigations because they have similar fluorescence lifetimes to that of HDPE plastic (Table 2). Owing to the variability of the samples, the presented results motivated further investigations to exploit the full potential of the FD-FLIM measurement method. The quantity of FD-FLIM data needs to be expanded for plastics and environmental materials, including the composition of soil, because our knowledge in these fields is currently restricted only to FD-FLIM data from our publications [25,26]. In addition to the published data, we are currently investigating the fluorescence properties of 13 types of plastics and 10 environmental materials, which is showing promising results [31]. These investigations can form the basis for directly identifying MPs in environmental matrices using FD-FLIM. Moreover, we can determine in which areas FD-FLIM can be a supporting analysis method combined with other analysis methods, such as micro-FTIR, Raman, or ATR spectroscopy. The limitations of the FD-FLIM method regarding identifiable sizes of MPs in environmental matrices must be addressed. In addition to determining these limitations, investigations on the impact of filler, additive, and dye concentrations on the fluorescence lifetime must be performed based on the numerous plastic compositions present in the environment. Future work must determine how biofilms influence the fluorescence lifetime exhibited by plastic types, and thus, whether the plastic can be directly identified when covered in a biofilm. Additionally, comparing FD-FLIM to current state-of-the-art methods, such as micro-FTIR, ATR, and Raman spectroscopy, is indispensable for highlighting the advantages and disadvantages of the method. Because this research using FD-FLIM to directly identify HDPE MPs in the environment was the first proof-of-principle study, further adjustments in measurement parameters and the applicability to detect other plastic types are currently being tested.

## Data availability statement

The data will be available by the authors upon request.

## CRediT authorship contribution statement

**Maximilian Wohlschläger:** Writing – review & editing, Writing – original draft, Visualization, Validation, Software, Project administration, Methodology, Investigation, Formal analysis, Data curation, Conceptualization. **Martin Versen:** Writing – review & editing, Validation, Supervision, Methodology, Investigation, Formal analysis. **Martin G.J. Löder:** Writing – review & editing, Validation, Supervision, Investigation, Formal analysis. **Christian Laforsch:** Writing – review & editing, Validation, Supervision, Investigation, Funding acquisition, Formal analysis.

## Declaration of competing interest

The authors declare that they have no known competing financial interests or personal relationships that could have appeared to influence the work reported in this paper.
